# Editorial: Clinical molecular biological characteristics of malignant tumors

**DOI:** 10.3389/fmolb.2026.1824594

**Published:** 2026-04-14

**Authors:** Xi Yang

**Affiliations:** 1 Huzhou Central Hospital, Fifth School of Clinical Medicine of Zhejiang Chinese Medical University, Huzhou, Zhejiang, China; 2 Huzhou Central Hospital, Affiliated Central Hospital Huzhou University, Huzhou, Zhejiang, China; 3 Zhejiang-France United Laboratory of Integrated Traditional Chinese and Modern Medicine in Colorectal Cancer, Huzhou, Zhejiang, China

**Keywords:** malignant tumors, molecular profiling, precision oncology, prognostic biomarkers, rare malignancies, tumor immune microenvironment

This Research Topic compiles six articles that investigate the molecular profiles and prognostic indicators across a spectrum of malignancies, ranging from prevalent carcinomas to rare sarcomas. The shared objective across these studies is to translate molecular and clinical data into actionable tools for patient stratification and targeted intervention.

In the context of biomarker discovery for common solid tumors, several contributions identified specific genetic and proteomic signatures linked to patient outcomes and the tumor immune microenvironment. Jiang et al. developed a 5-gene prognostic model based on disulfidptosis-related genes in colon cancer, demonstrating its utility in predicting overall survival and reflecting immune infiltration characteristics. For non-small cell lung cancer, Podemska et al. reported that MRPL23 overexpression serves as an independent prognostic factor for shorter overall survival, particularly within the squamous cell carcinoma subtype. Similarly, Jiang et al. established E2F2 as a risk factor in serous ovarian cancer, linking its elevated expression to poor prognosis, specific immune cell recruitment, and altered drug sensitivity.

Beyond common epithelial tumors, this Research Topic addresses the molecular characterization of rare and complex malignancies. Wei et al. provided a comprehensive mass spectrometry-based proteomic analysis of low-grade undifferentiated spindle cell sarcoma (USCS). By identifying differentially expressed proteins such as PHRF1 and DIDO1, their work offers baseline data for potential therapeutic targets in a disease that currently lacks specific predictive markers. Expanding on the complexity of rare clinical presentations, Xu et al. detailed a case involving the coexistence of low-grade pulmonary mucinous epithelioid carcinoma and metastatic adrenal sarcomatoid carcinoma, highlighting the critical role of the BRAF p.V600E mutation in tracking metastatic progression and guiding personalized targeted therapy.

Finally, the integration of clinical parameters into predictive models is addressed by Bai et al., who constructed a multiple linear regression model to predict the preoperative peritoneal cancer index in patients with pseudomyxoma peritonei. This approach utilizes standard preoperative variables to estimate surgical disease burden.

Together, these studies illustrate the ongoing transition from broad histopathological classification to precise molecular and clinical profiling, providing specific data points to optimize risk assessment and individualized treatment strategies.

